# Optimization of controlled-release fertilizer and urea ratio to increase maize yield by improving dry matter accumulation and translocation in black soil

**DOI:** 10.3389/fpls.2026.1820916

**Published:** 2026-06-12

**Authors:** Chunyan Yin, Liang Feng, Xiaoyu Liu, Yu Li, Xin Shen, Guohai Fu, Ju Zhao, Lele Tian, Qingmei Li

**Affiliations:** 1 Institute of Resources, Environment and Sustainable Development, Inner Mongolia Academy of Agricultural and Animal Husbandry Sciences, Hohhot, China; 2 Inner Mongolia Autonomous Region Industrial Technology Engineering Center for Efficient Utilization of Water and Soil Resources, Inner Mongolia Academy of Agricultural and Animal Husbandry Sciences, Hohhot, China; 3 National Agro-Tech Extension and Service Center, Beijing, China; 4 College of Agriculture, Inner Mongolia Agricultural University, Hohhot, China

**Keywords:** controlled-release fertilizer, dry matter accumulation, dry matter translocation, maize, nitrogen management, urea

## Abstract

Nitrogen is the principal mineral nutrient limiting maize yield formation and effective nitrogen management is thus key for maximizing yield. However, conventional urea releases nutrients rapidly and the supply of nutrients is often mismatched with crop demand, leading to early-stage nutrient loss and late-stage nitrogen deficiency. Therefore, reasonable nitrogen management is a key measure for increasing maize yield. This study aimed to evaluate the effects of different ratios of controlled-release fertilizer (CRF) and urea on changes in the accumulation, distribution, and translocation of dry matter across the entire growth period of maize to optimize nitrogen management, yield, and maize production efficiency. A two-year field experiment (2023–2024) was performed to test the effects of different CRF-N to urea-N ratios [1:0 (N10, controlled-release fertilizer), 3:7 (N37), 5:5 (N55), 7:3 (N73), and 0:1 (N01, conventional urea), no N (N0) as control] on dry matter accumulation, distribution, translocation and yield. The results showed that dry matter accumulation followed a typical sigmoidal (“S-shaped”) trajectory. The dry matter accumulation advantage of N73 (70% CRF) treatment was most significant after the V6 stage, and dry matter accumulation at R6 increased by 14.58% and 10.33% compared with that in N10 treatment in 2023 and 2024, respectively. With the progress of growth period, the distribution of dry matter among organs was changed, and N73 treatment effectively promoted dry matter allocation to the ear. Logistic equation fitting indicated that N73 treatment moderately delayed the time of the maximum dry matter accumulation rate ( $T_{max}$), sustained a relatively high maximum dry matter accumulation rate ( $V_{max}$), and extended the active growth period (*P*). In addition, N73 treatment increased both pre-anthesis dry matter remobilization (DMR) and post-anthesis assimilation (DMA), enhanced the contribution of dry matter accumulation to grain at post-anthesis (DMAC) and maize yield. In summary, a 7:3 CRF:urea application ratio optimizes maize dry matter accumulation and partitioning and coordinates pre-anthesis reserve remobilization with post-anthesis photosynthetic assimilation, and ultimately increased maize yield. Our findings have implications for optimizing nitrogen management, enhancing maize yield, and improving the efficiency of maize production. These results indicate that in the black soil region of Northeast China, a mixture of 70% controlled-release fertilizer and 30% urea is an effective strategy for promoting dry matter accumulation and transport, and for increasing yield of maize.

## 1 Introduction

Maize is an economically significant crop as a source of food, feed, and industrial raw materials, and achieving high, stable yields is vital for ensuring national food security (Zou et al., 2024). The black soil in Northeast China is a precious soil resource in China, known as the “giant panda in arable land”. It is the major maize producing area and the core guarantee area for food security in China. The black soil region has a large maize planting area and high yield level, occupying a strategic position in the national grain production pattern (Han and Zou, 2021; Li et al., 2022). Nitrogen is the principal mineral nutrient limiting maize yield formation, and effective nitrogen management is thus key for maximizing yield (Chen et al., 2011; Du et al., 2024). However, in order to pursuit high yield, problems have existed in maize cultivation, such as excessive application of nitrogen fertilizer, traditional urea single application, unreasonable fertilization during the growth period of corn (Yu et al., 2016), which ultimately decreases nitrogen use efficiency and can cause environmental problems (Wang et al., 2024). Consequently, developing efficient nitrogen management strategies is key for ensuring sustainable increases in maize yield.

Controlled-release fertilizers (CRF) regulate the nutrient release via coating and related technologies, which ensures that the requirements of nutrients are matching with fertilizer supply during crop growth, thereby improving nitrogen use efficiency and reducing losses (Gao et al., 2015; Li et al., 2025). However, relying solely on CRF can result in an insufficient nitrogen supply at the seedling stage and may increase costs (Li et al., 2021). Conventional urea releases nutrients rapidly, and can supply nutrients at crop seedling stage. Therefore, combining CRF with conventional urea can integrate the advantages of rapid and slow nutrient release and meet the nitrogen needs of maize throughout the entire growth cycle (Xiao et al., 2024; Guo et al., 2017).

The characteristics of dry matter accumulation, translocation, and distribution determine the source-sink balance and yield formation in maize (Chen et al., 2023; Shi et al., 2024; Yan et al., 2022). Crop yield depends on both the translocation of pre-anthesis reserves from vegetative organs to the grain and the direct assimilation of photosynthates after anthesis (Chen et al., 2023; Yan et al., 2022; Shi et al., 2024). Nitrogen management strongly regulates changes in dry matter by influencing plant nitrogen metabolism and photosynthetic performance (Du et al., 2024; Yan et al., 2024). Previous studies have shown that optimizing nitrogen application can significantly enhance dry matter accumulation and improve its allocation to economically significant organs, and thereby increase crop yield (Li et al., 2024; Ma et al., 2017). The combined application of controlled-release fertilizer and urea can effectively promote the growth of maize and increase grain yield (Guo et al., 2024; Zhang et al., 2026). Conventional urea leads to nitrogen supply-demand imbalance, insufficient dry matter accumulation, and low transport efficiency, which are key factors limiting yield and benefits in maize production in the black soil region of Northeast China. However, current research on the effects of different application ratios of controlled-release fertilizer to urea on the characteristics of dry matter accumulation and distribution in maize remains insufficient in the black soil region of Northeast China, especially lacking systematic analysis of dry matter translocation before and after flowering and its quantitative contributions.

In this study, we explored the effect of optimizing the ratio of controlled-release fertilizer to urea on dry matter accumulation, distribution, and translocation during maize growth through a two-year field experiment in the black soil region of Northeast China. Our aims were to 1) investigate the effects of optimizing the ratio of controlled-release fertilizer to urea on the characteristic of dry matter accumulation, distribution, and pre- and post-anthesis translocation; 2) identify the optimal nitrogen combination to maximize the production and efficiency of maize cultivation; and 3) elucidate the main determinants governing maize yield.

## 2 Materials and methods

### 2.1 Experimental site

**Figure 1:**
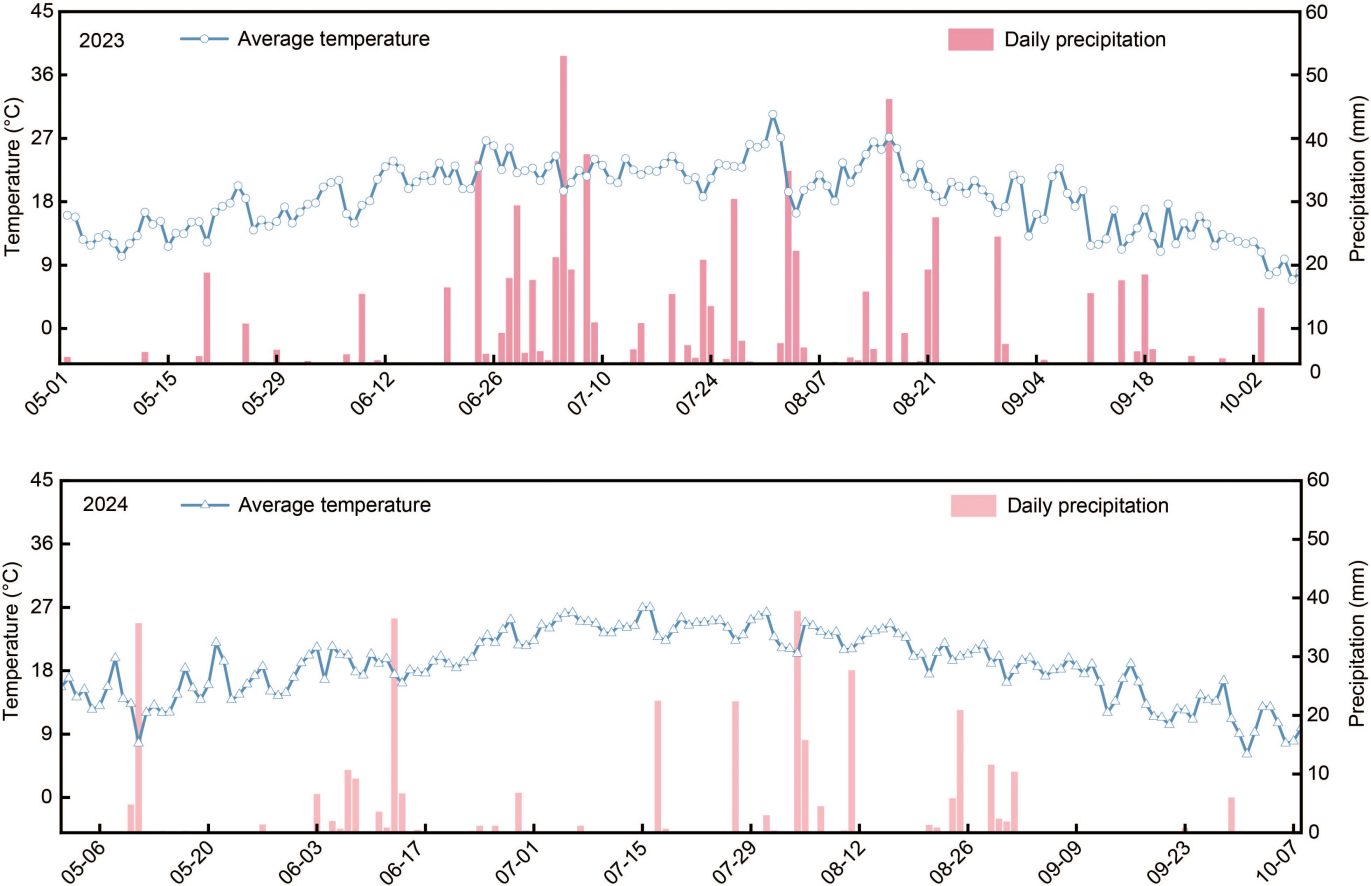
Maximum and minimum temperature and precipitation data for the study site during the growing season in 2023 and 2024. Reproduced from Tian et al., 2026, licensed under CC BY 4.0.

The experiment was conducted from May 2023 to October 2024 at the experimental base of the Arong Banner Agricultural Development Service Center, Hulunbuir City, Inner Mongolia Autonomous Region, China (48°09′21″N, 123°28′39″E). This area, with an altitude of 232.8 m, belongs to a temperate continental semi-humid climate. It is characterized by a mean annual temperature of 1.7 °C, a mean annual sunshine duration of 2800 h, an annual accumulated temperature (≥10 °C) of approximately 2400 °C, a mean annual precipitation of 450 mm, a mean annual evaporation of 1455 mm, and a frost-free period of 120 days. The precipitation distribution and temperature conditions during the experimental period are shown in **Figure 1**, and the main precipitation was concentrated from June to August. The soil is classified as black soil with organic matter 24.76 g/kg, total nitrogen 2.17 g/kg, alkali-hydrolyzable nitrogen 247.2 mg/kg, available phosphorus 16.47 mg/kg, available potassium 247.6 mg/kg, and pH 6.54 (Tian et al., 2026).

### 2.2 Experimental design

The experiment was performed in a randomized complete block design with six nitrogen treatments: CRF alone (controlled-release fertilizer, N10), urea alone (conventional fertilization, control, N01), CRF:urea N ratios [3:7 (N37), 5:5 (N55), 7:3 (N73)], and no fertilizer (N0, control). Each treatment had three replicates (18 plots total). The total N application rate was identical across treatments (285 kg N/ha). The application rates of slow-release fertilizer and urea for each treatment are shown in **Table 1**. Each plot measured 39 m^2^, with ridge planting at 65 cm row spacing and a target density of 75,000 plants/ha. The tested maize variety was Fengyu 8, supplied by the Arong Banner Agricultural Development Center. Fertilizers included urea (46% N), coated controlled-release fertilizer (40% N, S-shaped release curve with a 90-day release period, Stanley Fertilizer Co., China), diammonium phosphate (18% N, 46% P_2_O_5_), and potassium chloride (60% K_2_O). All other field management procedures followed standard local practices.

**Table 1:** Proportion of CRF and urea in each treatment.

Treatment	Fertilizer ratio	Proportion	CRF (kg/ha)	Urea (kg/ha)	Nitrogen fertilizer cost (yuan/ha)
N10	CRF only	1:0	712.5	0	2850.00
N37	CRF, Ure	3:7	213.75	433.70	2069.35
N55	CRF, Ure	5:5	356.25	309.78	2292.39
N73	CRF, Ure	7:3	498.75	185.87	2515.42
N01	Urea only	0:1	0	619.57	1734.78
N0	No fertilizer	0	0	0	0

According to the fertilizer market price in 2023-2024, CRF 4.0 yuan/kg, Urea 2.53 yuan/kg.

### 2.3 Measurement indicators and methods

At the V3 (seedling), V6 (jointing), VT (tasseling), R3 (grain-filling), and R6 (maturity) stages, 10 representative plants were randomly selected from each plot. Samples were separated into stems, leaves, and ears, fresh weights were recorded, and tissues were oven-heated at 105 °C for 30 min to inactivate enzymes, and subsequently dried at 75 °C to a constant weight, and measured dry weights of each organ (Dong et al., 2024). Additionally, the dry matter accumulation, translocation, contribution rates, dry matter remobilization at pre-anthesis (DMR, kg/ha), dry matter remobilization efficiency at pre-anthesis (DMRE, %), contribution of dry matter remobilization to grain at pre-anthesis (DMRCG, %), dry matter accumulation at post-anthesis (DMA, kg/ha), dry matter remobilization efficiency at post anthesis (DMAE, %), contribution of dry matter accumulation to grain at post anthesis (DMAC, %) were calculated (Wang et al., 2024).


$$DMR=NDM_{F}-NDM_{M}$$

$$DMRE=(NDM_{F}-NDM_{M})/NDM_{F}\times 100\%$$

$$DMRCG=(NDM_{F}-NDM_{M})/DM_{G}\times 100\%$$

$$DMA=DM_{M}-DM_{F}$$

$$DMAE=DMA/DM_{M}\times 100\%$$

DMAC=1−DMRCG

Where, $NDM_{F}$ is the dry matter accumulation in vegetative organs at flowering; $NDM_{M}$ is the dry matter accumulation in vegetative organs at maturity; $DM_{G}$ is the grain dry matter accumulation at maturity; $DM_{F}$ is the post-anthesis dry matter assimilation amount; $DM_{M}$ is the dry matter accumulation at maturity.

Logistic equation $y=a/(1+b\times e^{-cx})$ was used to fit the process of dry matter accumulation aboveground in maize. In the equation, x is the days after sowing, y is the dry matter accumulation, a is the maximum dry matter accumulation amount, b is the initial parameter, c is the growth rate parameter. Then calculate the maximum dry matter accumulation rate ( $V_{max}$), time of maximum accumulation rate ( $T_{max}$), and active growth period ( P) (Yang et al., 2024).


$$V_{max}=-ac/4$$

$$T_{max}=-\ln b/c$$

P=6/c

At R6 stage, each plot randomly selects two rows of 10-meter-long plants for yield measurement. After harvest, spike lengths, kernel numbers per spike, 1000-seed weights, and yields were measured. The yield of each plot was then calculated and converted into kg/ha.

### 2.4 Statistical analysis

We conducted one-way ANOVA at *P* < 0.05 to investigate the effects of different treatments on dry matter accumulation, dry matter in different maize organs, dry matter translocation. Spearman’ correlation coefficient analysis was performed between maize yield and dry matter accumulation in different growth stages, DMR, DMRE, DMRCG, DMA, DMAE, DMAC. We conducted these analyses using IBM SPSS Statistics 22.0. The figures were conducted in Origin 2025.

## 3 Results

### 3.1 Dry matter accumulation

**Figure 2:**
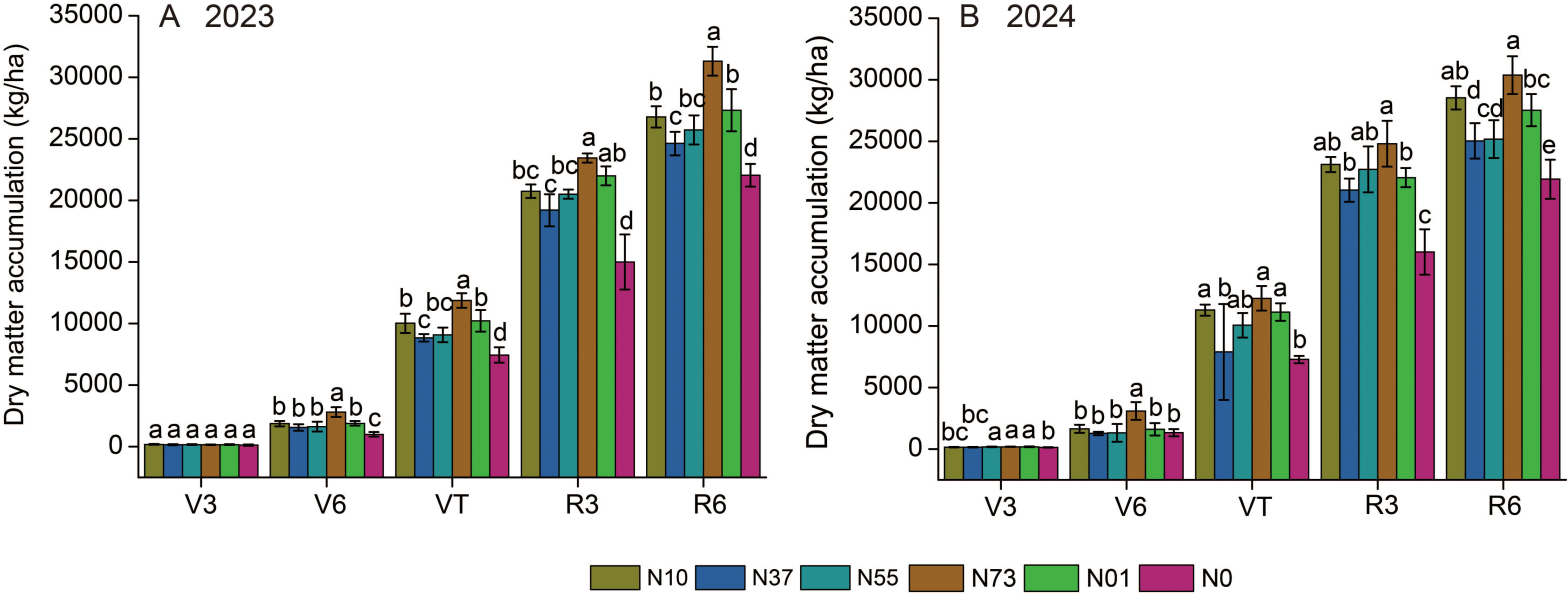
Effect of different application ratios of CRF and urea on the dry matter accumulation of maize in 2023 **(A)** and 2024 **(B)** growing seasons. N10, controlled-release fertilizer; N37, CRF:urea N ratios 3:7; N55, CRF:urea N ratios 5:5; N73, CRF:urea N ratios 7:3; N01, urea alone (conventional fertilization); N0, no fertilizer (control). Different letters indicate significant differences among treatments at the *P* < 0.05 level. Error bars are standard errors of mean (n = 3). V3, seedling stage; V6, jointing stage; VT, tasseling stage; R3, grain-filling stage; R6, maturity stage.

CRF:Urea ratios significantly affected the dry matter accumulation of maize, and N73 treatment had the highest dry matter accumulation in 2023 and 2024, respectively (**Figure 2**). In 2023, treatments differed significantly at multiple stages. N73 treatment showed a clearest advantage compared with other treatments as growth progressed, except at the seedling stage. Compared with the N01 treatment, the dry matter accumulation in N73 treatment increased 48.13%, 16.05%, 6.58%, and 14.58% at the V6, VT, R3 and R6 stages, respectively (**Figure 2A**, *P* < 0.05). In 2024, the changes of dry matter accumulation among treatments were basically consistent with that in 2023. Compared with the N01 treatment, the dry matter accumulation in N73 treatment increased 92.38%, 10.08%, 4.86%, and 10.33% at the V6, VT, R3 and R6 stages, respectively (**Figure 2B**, *P* < 0.05). N0 treatment consistently had the lowest values during the entire growth period of maize in both 2023 and 2024. In addition, during the entire growth period of maize, dry matter accumulation in all treatments exhibited an “S-shaped” growth trend with slow before V6, rapid after V6, and slowing again during R3 in both 2023 and 2024 (**Supplementary Figure 1**).

### 3.2 Distribution of dry matter in different maize organs

Dry matter accumulation is the foundation of crop yield, and investigating the distribution of dry matter among various organs can optimize the process of crop yield formation. From V3 stage to R6 stage, the ratio of leaf distribution was gradually decreasing; the ratio of spike distribution was gradually increasing, and reaching its maximum at R6 stage; while the ratio of stem distribution showed a trend of increasing first and then decreasing, with the highest ratio observed during the VT stage (**Supplementary Figure 2**).

**Figure 3:**
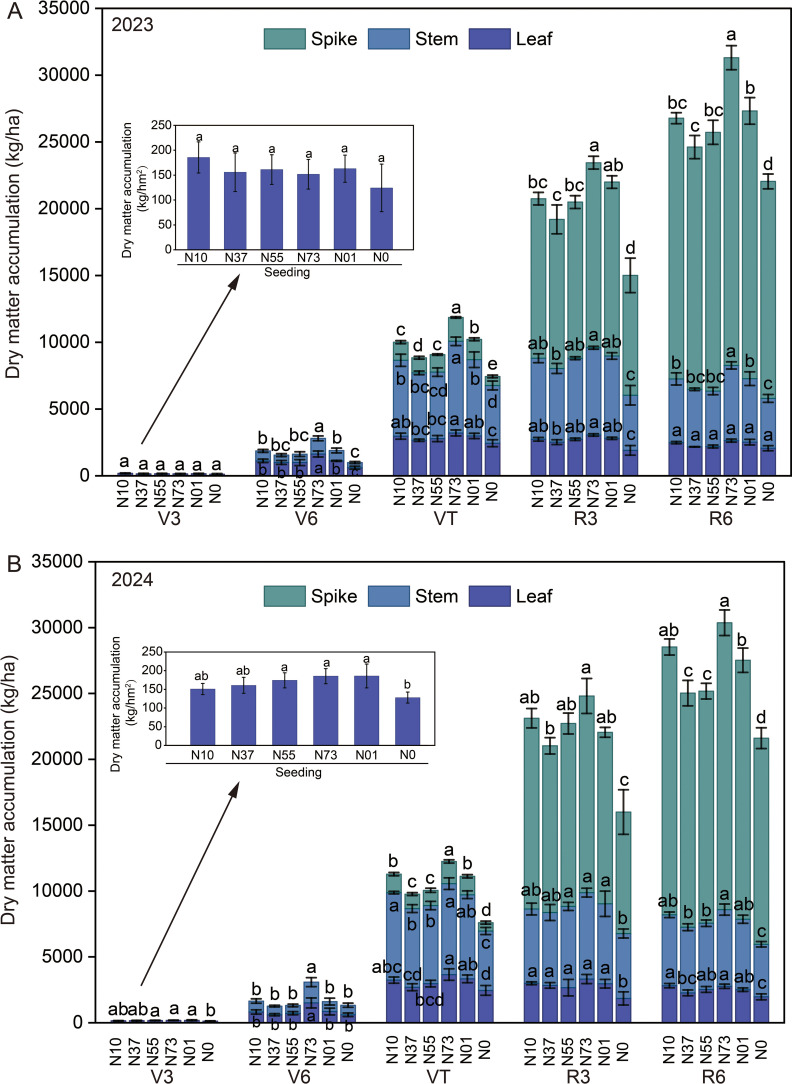
Effect of different CRF:urea ratios on dry matter accumulation in different organs in different stages in 2023 **(A)** and 2024 **(B)** growing seasons. N10, controlled-release fertilizer; N37, CRF:urea N ratios 3:7; N55, CRF:urea N ratios 5:5; N73, CRF:urea N ratios 7:3; N01, urea alone (conventional fertilization); N0, no fertilizer (control). Different letters indicate significant differences among treatments at the *P* < 0.05 level. Error bars are standard errors of mean (n = 3). V3, seedling stage; V6, jointing stage; VT, tasseling stage; R3, grain-filling stage; R6, maturity stage.

In addition, the ratios of CRF:Urea influenced the distribution of dry matter in different maize organs. At V6 stage, the dry matter accumulation in leaves and stems with N73 treatment significantly higher than that in other treatments (*P* < 0.05) in 2023 (**Figure 3A**) and 2024 (**Figure 3B**). At VT stage, the dry matter accumulation in leaves with N73 treatment significantly higher than that in N37, N55, and N0 treatments (*P* < 0.05) in 2023 (**Figure 3A**) and 2024 (**Figure 3B**), while there were no significant differences between N73, N10, and N01 treatment (**Figure 3**). The dry matter accumulation in stems and spikes with nitrogen treatment were significantly higher than that in N0 treatment, with the N73 treatment being the highest, followed by N01 and N10 treatment in both 2023 and 2024 at VT stage. Compared with N0 treatment, dry matter accumulation in stems and spikes with N73 treatment increased by 58.92% and 161.65% in 2023 (*P* < 0.05, **Figure 3A**), and 53.24% and 164.95% in 2024 (*P* < 0.05, **Figure 3B**), respectively. Compared with N01 treatments, dry matter accumulation in stems and spikes with N73 treatment increased by 20.26% and 17.44% in 2023 (P<0.05, **Figure 3A**), and 8.14% and 14.69% in 2024 (*P* < 0.05, **Figure 3B**), respectively. At R3 stage, the dry matter accumulation in leaves and stems with nitrogen treatments significantly higher than that in N0 treatment, but there were no significant differences between different CRF:Urea ratio treatments in 2023 (*P* < 0.05, **Figure 3A**) and 2024 (*P* < 0.05, **Figure 3B**), except for N37 treatment, which was significantly lower than N73 treatment in 2023. The dry matter accumulation in spike with nitrogen treatments significantly higher than that in N0 treatment, the performance among treatments was as follows N73 > N01 > N10, N55>N37 in 2023 (*P* < 0.05, **Figure 3A**) and N73 > N01, N10, N55>N37 in 2024 (*P* < 0.05, **Figure 3B**) at R3 stage. At R6 stage, the dry matter accumulation in stems and spikes with nitrogen treatments significantly higher than that in N0 treatment, except for N37 and N55 treatments, which showed no significant differences as compared with N0 treatment in 2023, and the performance among treatments was as follows N73 > N01, N10, N55, N37 in stems and N73 > N01, N10, N55>N37 in spike (*P* < 0.05, **Figure 3A**). In 2024, the dry matter accumulation in leaves with nitrogen treatments significantly higher than that in N0 treatment (*P* < 0.05), except for N37 treatment, which showed no significant difference as compared with N0 treatment (**Figure 3B**). The dry matter accumulation in stems and spikes with nitrogen treatments significantly higher than that in N0 treatment in 2024, and the performance among treatments was as follows N73, N01, N10 > N55, N37 in stems and N73, N10 > N01 >N37, N55 in spike (*P* < 0.05, **Figure 3B**).

### 3.3 Dry matter translocation

**Figure 4:**
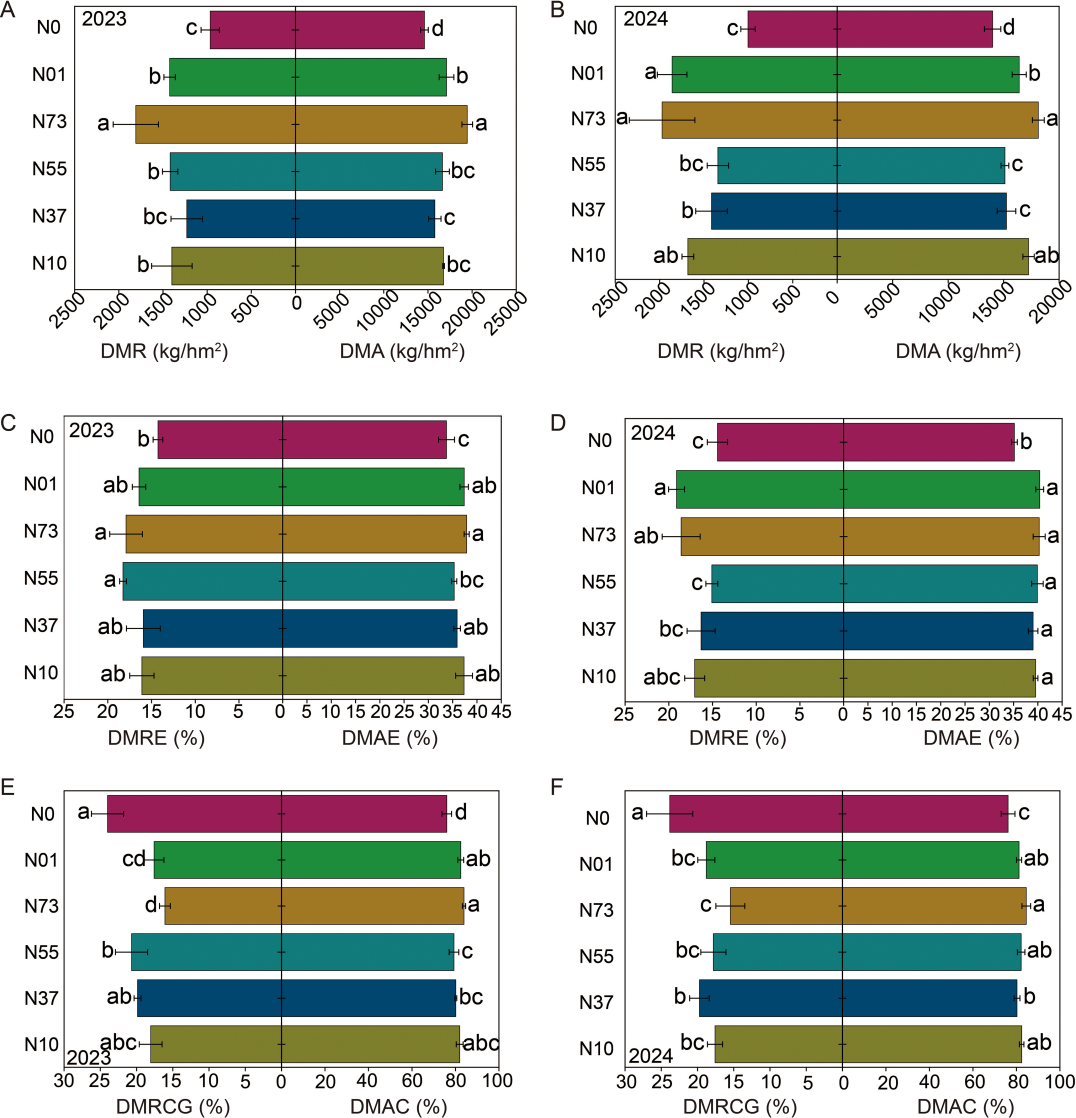
Changes in maize dry matter translocation under different CRF:urea ratios in 2023 and 2024 growing seasons. Different letters indicate significant differences among treatments at the *P* < 0.05 level. Error bars are standard errors of mean (n = 3). N10, controlled-release fertilizer; N37, CRF:urea N ratios 3:7; N55, CRF:urea N ratios 5:5; N73, CRF:urea N ratios 7:3; N01, urea alone (conventional fertilization); N0, no fertilizer (control). DMR, dry matter remobilization at pre-anthesis; DMRE, dry matter remobilization efficiency at pre-anthesis; DMRCG, contribution of dry matter remobilization to grain at pre-anthesis; DMA, dry matter accumulation at post-anthesis; DMAE, dry matter remobilization efficiency at post-anthesis; DMAC, contribution of dry matter accumulation to grain at post-anthesis. Different letters indicate significant differences among treatments at the *P* < 0.05 level. **(A)** DMR and DMA in 2023, **(B)** DMR and DMA in 2024, **(C)** DMRE and DMAE in 2023, **(D)** DMRE and DMAE in 2024, **(E)** DMRCG and DMAC in 2023, **(F)** DMRCG and DMAC in 2024. Error bars are standard errors of mean (n = 3).

Combining CRF with urea significantly increased DMR, DMA, DMRE, DMAE, DMAC, and reduced DMRCG compared with N0 in both 2023 and 2024 (**Figure 4**). N73 treatment caused a highest DMR and DMA in 2023, compared with other treatments, increased by 26.88%~87.21% and 13.71%~33.20%, respectively (*P* < 0.05, **Figure 4A**). Also, N73 treatment increased DMRE and DMAE in 2023, increased by 25.59% and 12.27% compared with N0 treatment (*P* < 0.05, **Figure 4C**), but there were no significant differences between different CRF:Urea ratio treatments, except for N55 treatment with a lower DMAE than 73 treatment (*P* < 0.05, **Figure 4C**). Moreover, DMRCG reduced by 16.00%~49.24% and DMAC increased by 1.81%~10.42% with N73 treatment compared to other treatments (*P* < 0.05, **Figure 4E**) in 2023, however, there were no significant differences between other treatments (**Figure 4E**). In 2024, DMR (**Figure 4B**) and DMRE (**Figure 4D**) was significant higher in N73 treatment than that in N55 and N37 (*P* < 0.05), but there were no differences between N73 and N10 and N01 treatments (**Figure 4B**). Also, DMA was significant higher with N73 treatment compared with other treatment, except for N10 treatment (*P* < 0.05, **Figure 4B**), which showed no difference with N73 treatment (**Figure 4B**). Furthermore, compared to N0, combining CRF with urea significantly increased DMAE (*P* < 0.05, **Figure 4D**), but there were no significant differences between different CRF:Urea ratio treatments. In addition, combining CRF with urea significantly decreased DMRCG and increased DMAC compared with N0 (*P* < 0.05, **Figure 4F**), N73 treatment showed lower DMRCG and higher DMAC compared with other treatments, but there was no significant difference between different CRF:Urea ratio treatments, except for N37 treatment.

### 3.4 Dynamics of dry matter accumulation in different organs of maize

Logistic equation $y=a/(1+b\times e^{-cx})$ was used to fit the process of dry matter accumulation aboveground in maize, and the determination coefficients R^2^>0.99 under different treatments (**Table 2**), indicated that Logistic equation can effectively simulate the dynamic changes in dry matter growth of maize. The results showed that the appropriate addition of CRF can delay the time of the maximum dry matter accumulation rate, increase the maximum dry matter accumulation rate, and extend the active growth period, thereby promoting dry matter accumulation and ultimately increasing maize yield (**Table 2**). In 2023, $T_{max}$ and P with N0 treatment was the longest, followed by N73 treatment, while $V_{max}$ was the shortest in N0 treatment and highest in N01 treatment, followed by N73 treatment. In 2024, $T_{max}$ and P with N0 treatment was the longest, while $V_{max}$ was the shortest in N0 treatment. Among all nitrogen treatments, $T_{max}$ was the highest in N37 treatment, followed by N01, N10, N73, and N55; $V_{max}$ was higher in N55 treatment, followed by N73, and lowest with N01 treatment; P was the highest in N37 treatment, followed by N01, N10, N37, and N55.

**Table 2:** Effects of different CRF:urea ratios on $T_{max}$, $V_{max}$, and P.

Year	Treatment	Growth curve parametric equation	Correlation coefficient	$T_{max}$	$V_{max}$	P
2023	N10	y=362.89/(1 + 402.59*exp(-0.06x))	0.9957958	92.52879226	5.880849587	92.56104933
	N37	y=333.34/(1 + 491.18*exp(−0.07x))	0.9964543	92.88322427	5.559850275	89.93337376
	N55	y=346.73/(1 + 626.97*exp(−0.07x))	0.997334	92.21106585	6.054710828	85.89901001
	N73	y=431.61/(1 + 246.20*exp(−0.06x))	0.9956262	94.04465077	6.317534818	102.4797361
	N01	y=366.22/(1 + 600.40*exp(−0.07x))	0.9969437	90.79296183	6.451331648	85.15041494
	N0	y=315.95/(1 + 245.42*exp(−0.05x))	0.9927	100.9326395	4.306480976	110.0490568
2024	N10	y=379.03/(1 + 1163.42*exp(−0.08x))	0.9941591	91.27742075	7.328300986	77.58258681
	N37	y=332.39/(1 + 2394.28*exp(−0.09x))	0.9969368	91.4876425	7.067318687	70.54843431
	N55	y=362.64/(1 + 25682.87*exp(−0.12x))	0.9983246	86.58334291	9.576227996	51.16422763
	N73	y=433.91/(1 + 895.68*exp(−0.08x))	0.9983246	90.51214523	8.14680238	79.89211632
	N01	y=366.07/(1 + 963.27*exp(−0.08x))	0.9930485	91.36099113	6.882136668	79.78739103
	N0	y=304.75/(1 + 455.86*exp(−0.06x))	0.9944003	98.34109723	4.725822955	96.72880053

*T_max* represents the time to maximum dry matter accumulation rate, *V_max* denotes the maximum rate of dry matter accumulation, and P indicates the active dry matter accumulation period.

### 3.5 Maize yield

**Figure 5:**
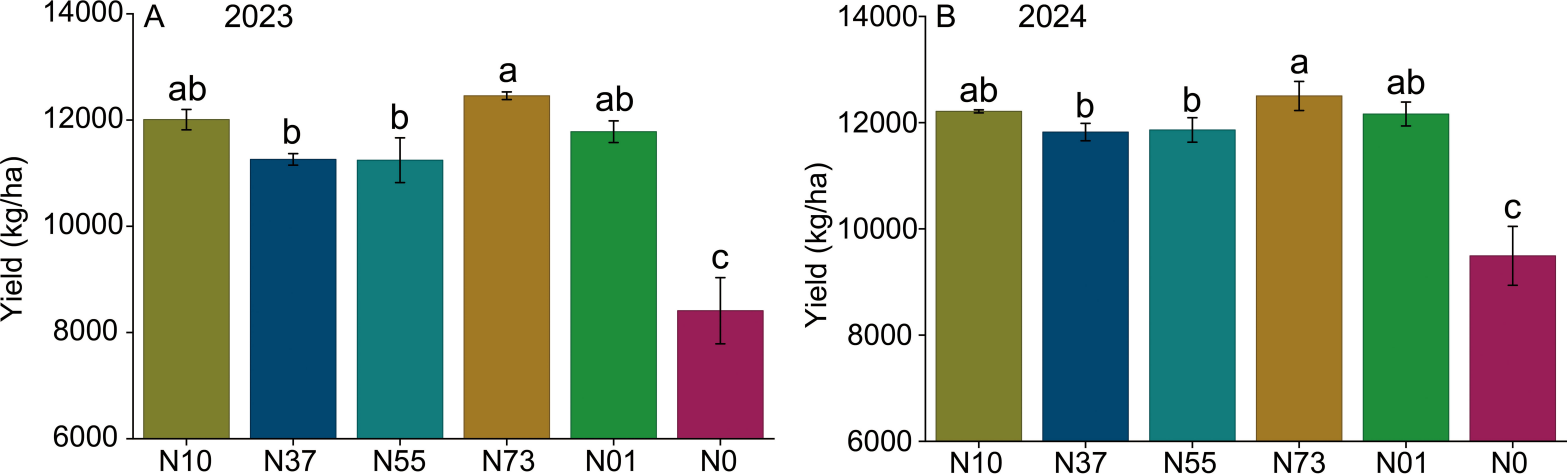
Changes in maize dry matter translocation under different CRF:urea ratios in 2023 **(A)** and 2024 **(B)** growing seasons. N10, controlled-release fertilizer; N37, CRF:urea N ratios 3:7; N55, CRF:urea N ratios 5:5; N73, CRF:urea N ratios 7:3; N01, urea alone (conventional fertilization); N0, no fertilizer (control). Different letters indicate significant differences among treatments at the *P* < 0.05 level. Error bars are standard errors of mean (n = 3).

The maize yield was significantly influenced by different CRF:Urea ratios (*P* < 0.05, **Figure 5**). The highest maize yield was achieved with N73, although there was no statistical significance when compared to treatments N10 and N01. In comparison to the N0 treatment, the maize yield varied from 33.69% to 48.13% in 2023 (**Figure 5A**), and from 24.55% to 31.72% in 2024 (**Figure 5B**) with different CRF:Urea ratio treatment. And the maximum maize yield to different CRF:Urea ratios was observed at the N73 treatment, reaching 12457.65 kg/ha in 2023 and 12502.92 kg/ha in 2024. The yield of maize in N01 treatment was 11780.45 kg/ha in 2023 and 12162.48 kg/ha in 2024. We calculated the economic benefits of different treatments, the results showed that nitrogen fertilizer cost in N01 and N73 treatments were 1734.78 and 2515.42 yuan/ha (**Table 1**). The prices of maize were 2.88 and 2.53 yuan/kg in 2023 and 2024, respectively. So compared with N01 treatment, the benefits in N73 treatment increased 1169.69 yuan/ha in 2023 and 80.68 yuan/ha in 2024.

**Figure 6:**
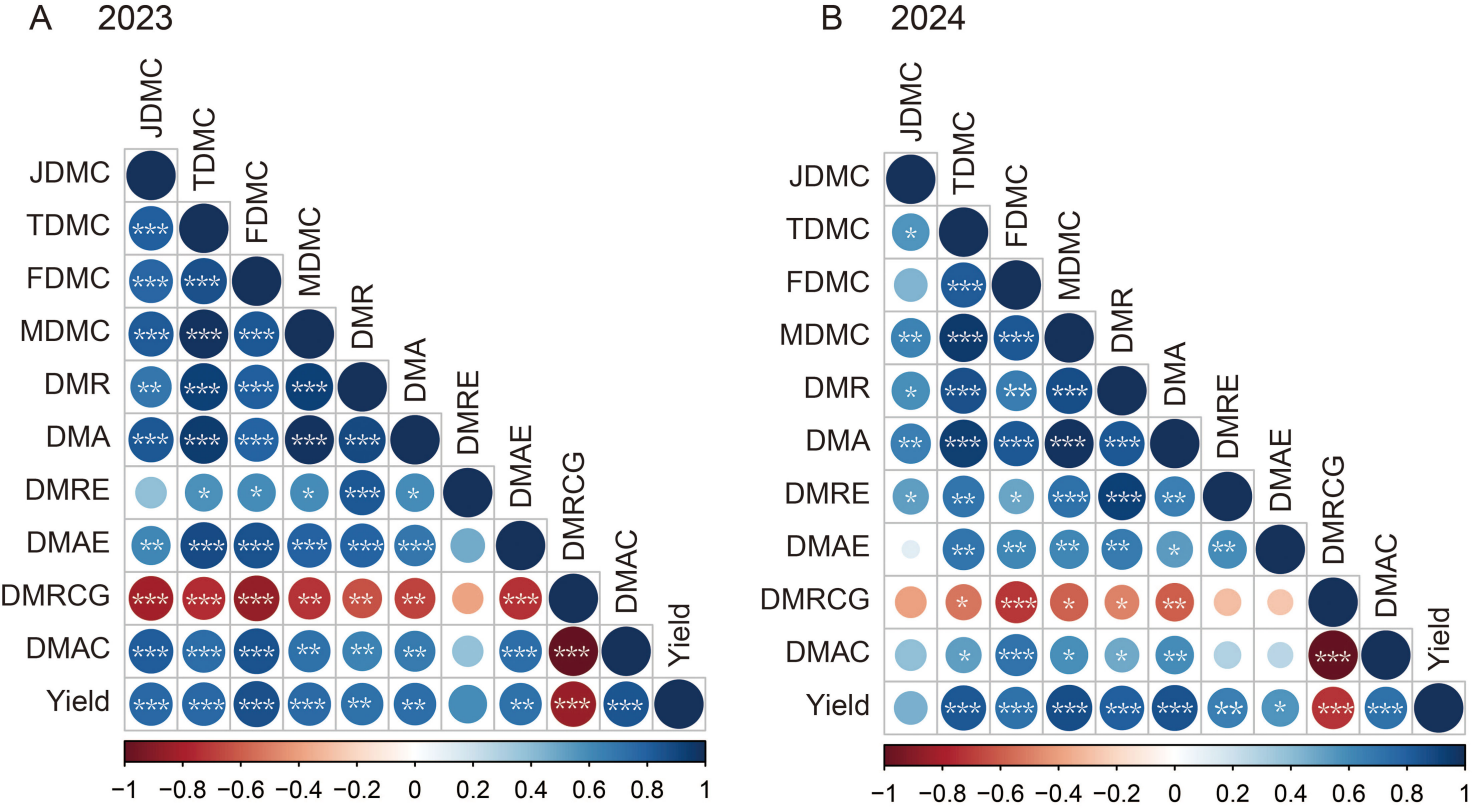
Correlation between maize yield and dry matter accumulation and translocation in 2023 **(A)** and 2024 **(B)** growing seasons. JDMC, dry matter accumulation at jointing stage; TDMC, dry matter accumulation at tasseling stage; FDMC, dry matter accumulation at filling stage; MDMC, dry matter accumulation at maturity stage; DMR, dry matter remobilization at pre-anthesis; DMA, dry matter accumulation at post-anthesis; DMRE, dry matter remobilization efficiency at pre-anthesis; DMAE, dry matter remobilization efficiency at post-anthesis; DMRCG, contribution of dry matter remobilization to grain at pre-anthesis; DMAC, contribution of dry matter accumulation to grain at post-anthesis.

Correlation analysis showed that JDMC, TDMC, FDMC, MDMC, DMR, DMA, DMAE, DMAC were significantly positively correlated with maize yield, and DMRCG was significantly negatively associated with maize yield in 2023 (**Figure 6A**, *P* < 0.01). In 2024, TDMC, FDMC, MDMC, DMR, DMA, DMRE, DMAE, DMAC were significantly positively correlated with maize yield, and DMRCG was significantly negatively associated with maize yield (**Figure 6B**, *P* < 0.05).

## 4 Discussion

### 4.1 Effect of the CRF:urea ratio on dry matter accumulation in maize

The accumulation and distribution of dry matter affect the process of maize yield formation (Hong et al., 2025). Optimizing nitrogen supply can regulate dry matter accumulation during the rapid and slow growth phases, promoting maize dry matter accumulation and, in turn, enhancing yield (Yang et al., 2025). In our study, we found that dry matter accumulation in maize followed a typical S-shaped growth curve (Yin et al., 2003), and the CRF:urea ratio significantly affected this process. N73 treatment (70% CRF-N + 30% urea-N) increased the dry matter accumulation and maintained a persistent advantage after the V6 stage, and achieved significantly greater dry matter accumulation at R6 stage compared with other treatments, including the conventional urea treatment (N01). This is consistent with previous studies that combined application of controlled-release and conventional nitrogen fertilizer significantly increased dry matter and nitrogen accumulation in summer maize, which initially increased and then decreased with the increasing proportion of controlled-release nitrogen fertilizer, and 75% controlled-release nitrogen fertilizer combined with 25% conventional nitrogen fertilizer synergistically improved nitrogen use efficiency and yield (Sun et al., 2024). This phenomenon primarily arises from the slow-release characteristics of CRF. By sustaining stable nitrogen supply in the middle and late growth stages, CRF sufficiently meets crop nutrient requirements during vigorous growth and grain filling, and prevents late-stage nitrogen deficiency associated with conventional urea fertilization (Li et al., 2024). Previous study found that the application of CRF improved soil nitrogen supply in the late growth stage of summer maize, maintained a higher net photosynthetic rate of leaves, and promoted the accumulation of above-ground dry matter and grain yield formation (Fan et al., 2015). Inappropriate CRF ratios produce distinct adverse effects, and low CRF ratios (e.g., N01, N37) may lead to late-stage nitrogen deficiency, while excessively high ratios (above N73) exhibit slow early nitrogen release, restricting early vegetative growth. Parameters of the Logistic model further demonstrated that the N73 treatment moderately delayed $T_{max}$​, sustained a relatively high maximum growth rate, and prolonged the effective growth duration. Such a steady dry matter accumulation pattern is critical to maize high-yield formation (Hou et al., 2015).

### 4.2 Effect of the CRF:urea ratio on the dry matter distribution in maize

The appropriate partitioning of dry matter among organs is essential for maximizing yield. With the progression of maize growth stages, the center of dry matter allocation shifted from leaves at the V3 stage to leaves and stems at V6, and ultimately to the spike during R3 and R6 stages, consistent with the source-sink transformation pattern of maize (Borrás and Westgate, 2006). In this study, N73 treatment enhanced dry matter accumulation in maize leaves, stems and grains. Meanwhile, the proportion and absolute quantity of dry matter partitioned to maize spikes were greater in the N73 treatment than other treatments at the VT, R3, and R3 stages. This may be attributed to the fact that combined application of CRF with urea altered leaf basal angle and specific light interception per unit leaf area, thereby improving light interception, photosynthetic capacity and dry matter production (Lai et al., 2025). Previous study demonstrated that controlled-release fertilizer extended the duration of large photosynthetic leaf area of summer maize, sustained stable photosynthesis after silking, enhanced post-silking dry matter accumulation, and retarded leaf senescence at grain filling, which facilitated the increase in grain weight and final yield (Guo et al., 2024). In addition, the amount and efficiency of nutrient translocation serve as crucial indicators for nutrient remobilization from vegetative organs to grains, thereby establishing a solid basis for maize yield enhancement (Xie et al., 2014; Yin et al., 2020). Our previous study showed that 70% CRU mixture with 30% urea application enhanced nitrogen absorption, utilization and translocation efficiency in maize, and promoted nitrogen remobilization from vegetative organs to grains (Tian et al., 2026), and thereby prolonged leaf functional longevity in later stages (strengthening the “source”) while simultaneously enhancing the establishment and filling capacity of the “sink” (spike) (Zhao et al., 2010). Overall, the N73 treatment effectively balanced vegetative and reproductive growth, facilitated photosynthate remobilization and allocation to grains, and optimized dry matter accumulation among leaves, stems and grains, thereby improving maize grain yield.

### 4.3 Effect of the CRF:urea ratio on dry matter translocation in maize

Pre-anthesis vegetative organ establishment and post-anthesis efficient dry matter accumulation jointly determine maize yield potential. Optimized management practices that guarantee adequate post-anthesis dry matter accumulation and strengthen pre-anthesis dry matter contribution to grain yield are key to improving maize yield and exploring its production potential (Latifmanesh et al., 2018; Wang et al., 2023). In this study, compared with N01, N73 significantly increased both pre-anthesis dry matter remobilization and post-anthesis dry matter assimilation and improved their contributions to grain accumulation. This is consistent with previous studies that combined application of CRU and conventional urea at an optimal ratio enhanced post-anthesis dry matter production and its supply for grain filling, thereby raising grain weight and final grain yield (Zheng et al., 2023). This may be because the combined application of CRU and conventional urea supplied appropriate nitrogen in the early period, enhancing dry matter partitioning to roots and promoting root growth as well as total root length. Subsequently, vigorous root systems with adequate late-stage nitrogen supply retarded leaf senescence, improved post-anthesis dry matter production and grain-filling supply, thus increasing grain weight and grain yield (Zheng et al., 2023). Analysis of variance further confirmed that the application ratio had significant or highly significant effects on key dry matter translocation indicators. A modest share of fast-acting urea (30%) met nitrogen demand from the seedling stage through the jointing stage, promoting vegetative growth and establishing the material basis for pre-anthesis accumulation and subsequent remobilization (greater pre-anthesis translocation). A high proportion of CRF (70%) sustained the nitrogen supply after anthesis and the leaf N accumulation under C70 reached 167.73 and 164.09 kg/ha in 2023 and 2024, respectively (Tian et al., 2026). This maybe benefit for leaf photosynthetic function and delayed senescence, and thereby increased post-anthesis assimilation and its contribution to the grain (Paponov and Engels, 2003). This pattern is a critical pathway for enhancing maize yields (Guo et al., 2017). Low CRF proportions may result in a deficient post-anthesis nitrogen supply and diminished photosynthetic capacity, whereas conventional urea can oversupply nitrogen before anthesis or deplete it rapidly after anthesis, which can preclude efficient dry matter translocation and partitioning. The mixed application of controlled-release urea and conventional urea (an optimal mixing ratio of 7:3) can achieve complementary effects between the slow-release characteristic of controlled-release nitrogen fertilizer and the rapid availability of conventional urea. It synchronizes nitrogen release with the nutrient demand pattern of maize, improves fertilizer use efficiency, enlarges grain sink capacity, promotes the translocation and allocation of photosynthate to grains, and ultimately achieves yield increase.

### 4.4 Effect of the CRF:urea ratio on maize yield

Previous studies have found that nitrogen fertilizer application can significantly improve crop yield (Guo et al., 2017; Sun et al., 2023). However, excessive nitrogen application not only fails to increase yield but also results in resource waste. Therefore, exploring a reasonable nitrogen application level is of great significance for reducing fertilizer use and increasing efficiency (Zhang et al., 2004). Slow-release nitrogen fertilizer can prolong the fertilizer effect, reduce nitrogen loss, and improve nitrogen fertilizer utilization rate (Hou et al., 2021). In this study, we found that both urea and controlled-release nitrogen fertilizer alone and in combination can improve maize yield compared to N0 treatment. In addition, the controlled-release nitrogen fertilizer and urea blending rates have different effects on maize yield (Ji et al., 2022). In our study, we found that maize yield and benefits within N73 treatment was higher than that in N01 treatment. This may be due to that this ratio of controlled-release urea to regular urea both meets the nutrient requirements of maize in the early stages of fertility and provides a stable supply of nutrients for growth in the middle and late stages of fertility. In this study, we found that JDMC, TDMC, FDMC, MDMC, DMR, DMA, DMAC were significantly positively correlated with maize yield, and DMRCG was significantly negatively associated with maize yield. In addition, N73 treatment optimized maize dry matter accumulation, translocation and nitrogen supply dynamics, and maintained stable grain yield, improved nitrogen use efficiency and achieved better economic benefits. Therefore, the combined application strategy of 70% controlled-release fertilizer and 30% conventional urea is suitable for maize production in the black soil region of Northeast China.

## 5 Conclusion

Our finding demonstrated that the efficacy of combined CRF and urea application in promoting maize dry matter accumulation, distribution, translocation and yield, and identified the N73 treatment as the optimal ratio for optimizing maize growth dynamics, dry matter allocation and yield. Specifically, N73 treatment established a dry matter accumulation pattern characterized by “stable initiation, high rate, and long duration” (as supported by logistic equation), and thus formed a “slowing down before and accelerating later” allocation strategy, which optimized the allocation of photosynthates to the ear, and achieved the concurrent enhancement of efficient pre-anthesis reserve remobilization and high post-anthesis photosynthetic assimilation, thus providing the most robust material basis for grain filling. And finally achieve the enhancement of crop yield and economic benefits. Overall, a 7:3 (N73) blend of controlled-release nitrogen fertilizer and urea is the optimal ratio for maximizing maize yield in the black soil region of Northeast China. This blend will help us optimize nitrogen fertilizer management and synergistically achieve high yield, high efficiency, and green development, which is of great theoretical and practical significance for protecting black soil resources and ensuring food security.

## Data Availability

The original contributions presented in the study are included in the article/**Supplementary Material**. Further inquiries can be directed to the corresponding author/s.
