# Supplementary material for: Optimization of controlled-release fertilizer and urea ratio to increase maize yield by improving dry matter accumulation and translocation in black soil
Source: Front Plant Sci. 2026 Jun 12;17:1820916. doi: 10.3389/fpls.2026.1820916 (PMC13308681; doi:10.3389/fpls.2026.1820916)
Supplement: Supplementary file 1 [file Table1.docx]

**Supplemental Material**

**Supplemental Figures**


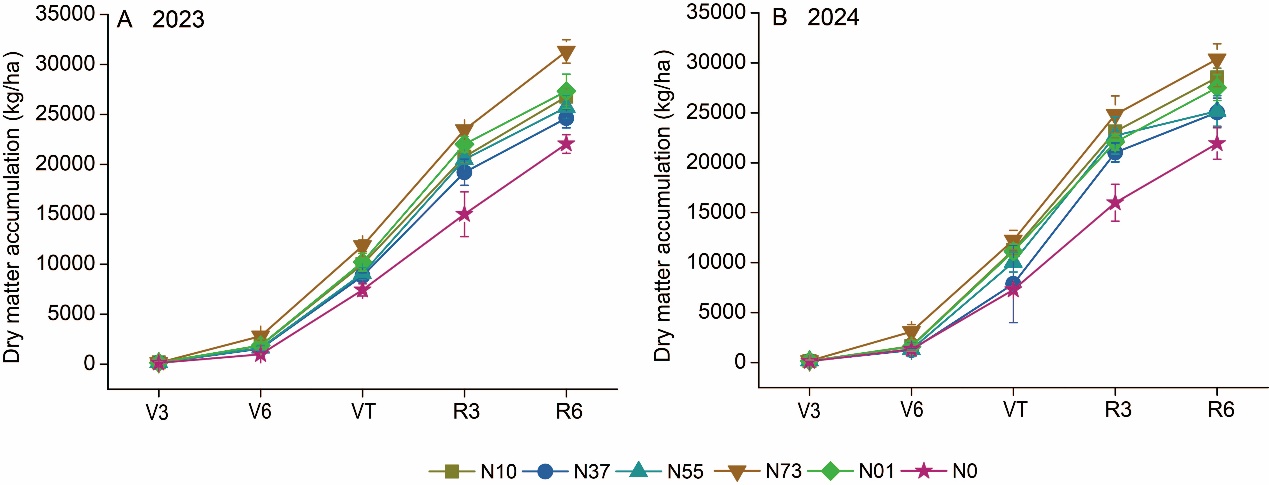


**Fig. S1.** Effect of different application ratios of CRF and urea on dry matter accumulation of maize. N10, controlled-release fertilizer; N37, CRF:urea N ratios 3:7; N55, CRF:urea N ratios 5:5; N73, CRF:urea N ratios 7:3; N01, urea alone (conventional fertilization); N0, no fertilizer (control).


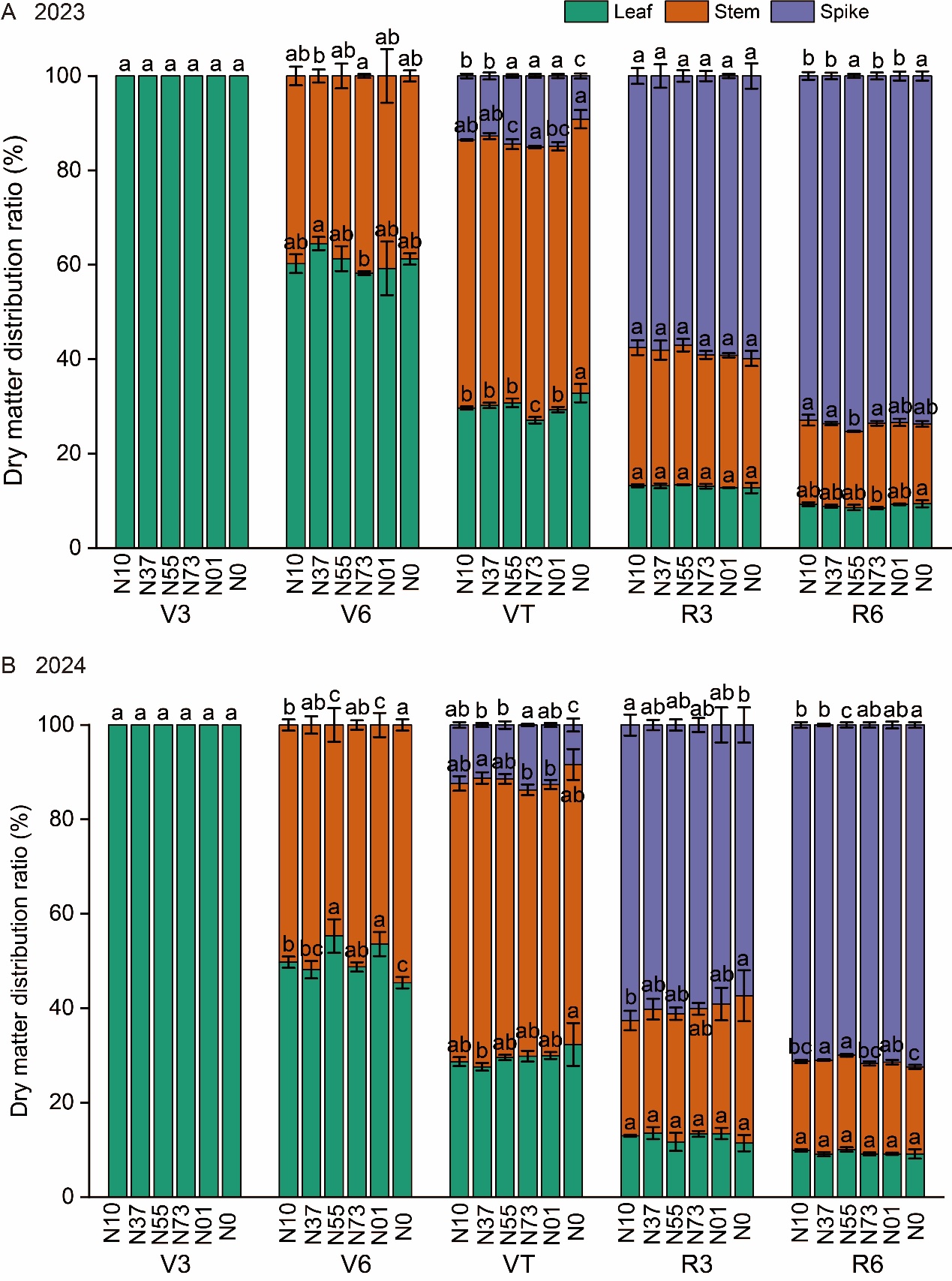


**Fig. S2** Effect of different application ratios of CRF and urea on the dry matter distribution ratio in maize in 2023 and 2024. N01, controlled-release fertilizer; N37, CRF:urea N ratios 3:7; N55, CRF:urea N ratios 5:5; N73, CRF:urea N ratios 7:3; N10, urea alone (conventional fertilization); N0, no fertilizer (control). Different letters indicate significant differences among treatments at the P < 0.05 level. Error bars are standard errors of mean (n = 3). V3, seedling stage; V6, jointing stage; VT, tasseling stage; R3, grain-filling stage; R6, maturity stage.
